# Cell density and airspace patterning in the leaf can be manipulated to increase leaf photosynthetic capacity

**DOI:** 10.1111/tpj.13727

**Published:** 2017-11-15

**Authors:** Christoph Lehmeier, Radoslaw Pajor, Marjorie R. Lundgren, Andrew Mathers, Jen Sloan, Marion Bauch, Alice Mitchell, Chandra Bellasio, Adam Green, Daniel Bouyer, Arp Schnittger, Craig Sturrock, Colin P. Osborne, Stephen Rolfe, Sacha Mooney, Andrew J. Fleming

**Affiliations:** ^1^ Department of Animal and Plant Sciences University of Sheffield Western Bank Sheffield S10 2TN UK; ^2^ Division of Agriculture and Environmental Sciences School of Biosciences University of Nottingham Sutton Bonington Campus Loughborough LE12 5RD UK; ^3^ Department of Physics and Astronomy University of Sheffield Hounsfield Road Sheffield S3 7RH UK; ^4^ Institut de Biologie Moleculaire des Plantes du CNRS IBMP‐CNRS‐UPR2357 12, rue du General Zimmer 67084 Strasbourg France; ^5^ Present address: Department of Ecology and Evolutionary Biology Kansas Biological Survey The University of Kansas 2101 Constant Ave. Lawrence KS 66047 USA; ^6^ Present address: Research School of Biology Australian National University Acton ACT 2601 Australia; ^7^ Present address: Institut de Biologie de l'ENS UMR8197 ‐ INSERM U1024 Ecole Normale Supérieure 46 rue d'Ulm 75230 Paris cedex 05 France; ^8^ Present address: Department of Developmental Biology University of Hamburg Biozentrum Klein Flottbek Ohnhorststr. 18 – 22609 Hamburg Germany

**Keywords:** leaf, cell division, photosynthesis, *Arabidopsis thaliana*, differentiation

## Abstract

The pattern of cell division, growth and separation during leaf development determines the pattern and volume of airspace in a leaf. The resulting balance of cellular material and airspace is expected to significantly influence the primary function of the leaf, photosynthesis, and yet the manner and degree to which cell division patterns affect airspace networks and photosynthesis remains largely unexplored. In this paper we investigate the relationship of cell size and patterning, airspace and photosynthesis by promoting and repressing the expression of cell cycle genes in the leaf mesophyll. Using microCT imaging to quantify leaf cellular architecture and fluorescence/gas exchange analysis to measure leaf function, we show that increased cell density in the mesophyll of Arabidopsis can be used to increase leaf photosynthetic capacity. Our analysis suggests that this occurs both by increasing tissue density (decreasing the relative volume of airspace) and by altering the pattern of airspace distribution within the leaf. Our results indicate that cell division patterns influence the photosynthetic performance of a leaf, and that it is possible to engineer improved photosynthesis via this approach.

## Significance Statement

The efficiency of photosynthesis is influenced by the distribution of cell material (where photosynthesis occurs) and airspace (by which the carbon dioxide for photosynthesis is delivered), yet the manner and degree to which cell division impacts this balance and, thus, photosynthesis remains largely unexplored. Using a transgenic approach, this paper demonstrates that it is possible to engineer increased photosynthesis by manipulating the patterns of cell division and airspace.

## Introduction

The internal architecture of a leaf arises by a combined process of cell growth and division, coupled to regulated cell separation events that determine the pattern and extent of airspace formed in particular regions of the leaf. This pattern of airspace, coupled with the spatial pattern of cell shapes, leads to the classically described palisade and spongy mesophyll histology of the typical angiosperm leaf. As a result of extensive investigations we now have detailed knowledge of the plant cell cycle and, as a consequence, access to a number of genes in which modulation leads to predictable changes in division rate and cell patterning, and therefore leaf morphology (Dewitte *et al*., 2003; Verkest *et al*., 2005a,b; De Veylder *et al*., 2007). In contrast, our understanding of the cell wall processes that lead to the controlled partial separation of cells subsequent to division (schizogeny) is much less advanced. Numerous enzyme activities (and associated genes) have the potential to modulate cell wall dissolution (McCann and Carpita, 2008), but how these activities are spatially and temporally co‐ordinated with cell division within the leaf remains unclear. Thus, although one might expect the patterning of intercellular airspaces to be altered as a consequence of altered cell size and packing, this downstream aspect of cell cycle gene manipulation has generally been overlooked, despite the fact that the core leaf physiological function of photosynthesis is likely to be significantly influenced (Flexas *et al*., 2008; Terashima *et al*., 2011). For example, altering cell size and packing is likely to alter the cell surface area available for gas diffusion, and the extent and tortuosity of the air channels linking photosynthetic cells to the stomata (which regulate the entry and exit of CO_2_ and water) will influence the diffusion of gases within the leaf. Although such structure–function relationships have long been recognized, they have generally involved correlating differences in leaf structure among different or related species rather than using molecular genetic techniques to manipulate structure within a species to identify causal relationships (Giuliani *et al*., 2013). We are interested in exploring the relationship between cell pattern and assimilatory physiology in the context of leaf development. Are there general rules relating cell division, growth and separation to the higher order architecture of the leaf, how do these rules relate to assimilatory physiology, and is there room for altering cell patterns to improve the efficiency of photosynthesis, a major target for crop improvement (Zhu *et al*., 2010)?

One reason for the lack of progress in this area has been the need for an easily implemented and robust methodology to detect and quantify leaf airspace in 3D. Classical approaches generally involve fixing tissue, taking sections, manually measuring airspace in these 2D sections and then estimating the volume of 3D airspace (John *et al*., 2013). These approaches are labour intensive, invasive and involve a number of assumptions about cell shape for the estimation of the values obtained. The advent of X‐ray micro‐computed tomography (microCT) and its implementation to plant sciences has provided a new method to both visualize and quantify airspace in a non‐invasive fashion (Cloetens *et al*., 2006; Dhondt *et al*., 2010). Living leaves can be scanned to generate 3D renderings of the internal architecture of the sample, easily distinguishing cellular material from the less dense airspace. These data sets can then be interrogated to derive various quantitative measures of airspace morphology, such as porosity, exposed surface area, channel size and, simultaneously, the arrangement of the tissues defining the airspace (Dorca‐Fornell *et al*., 2013; Pajor *et al*., 2013). Moreover, the analysis can be delimited to specific regions of interest within the organ, thus enabling the exploration of potential linkages of local structure to whole organ function.

Variability in leaf airspace is not directly under genetic control; rather, it is an emergent property related to cell division, size and separation, and the consequences these have on cell packing. At present, the best tools we have to influence this packing (and thus intervening airspace) are various components of the plant cell cycle (De Veylder *et al*., 2007). Manipulation of these genes leads to changes in both cell size and cell packing. For example, numerous publications have shown that overexpression of *KIP‐RELATED PROTEIN 1* (*KRP1*) tends to decrease proliferation, leading to larger cells (Dewitte *et al*., 2003; Verkest *et al*., 2005a,b; Weinl *et al*., 2005; Kuwabara *et al*., 2011). Conversely, the suppression of *RETINOBLASTOMA RELATED PROTEIN 1 (RBR1)* can be used to generate organs with smaller cells (Wildwater *et al*., 2005) and, indeed, in a previous paper we reported on the establishment of a combined microCT/chlorophyll fluorescence and gas exchange analytical approach for Arabidopsis using an inducible RBR RNAi transgenic line as a test case (Dorca‐Fornell *et al*., 2013). The uniform expression of the construct in this transgene led to changes in epidermal characteristics (notably stomatal density and associated stomatal conductance), however, which confounded a clear interpretation of the outcome on mesophyll differentiation and function.

In this paper we set out to generate Arabidopsis leaves in which mesophyll cell proliferation was either promoted or repressed (using both *KRP1* and *RBR1* constructs) in order to investigate the outcome of resultant increased and decreased cell size on airspace pattern, and the consequence of such altered cell/airspace pattern on photosynthetic performance. Our data show that the generation of more, smaller cells in the mesophyll can lead to an increase in leaf photosynthetic capacity and implicate airspace pattern as an important factor in this process.

## Results

### The modulation of cell cycle gene expression leads to altered leaf cellular architecture

**Figure 1:**
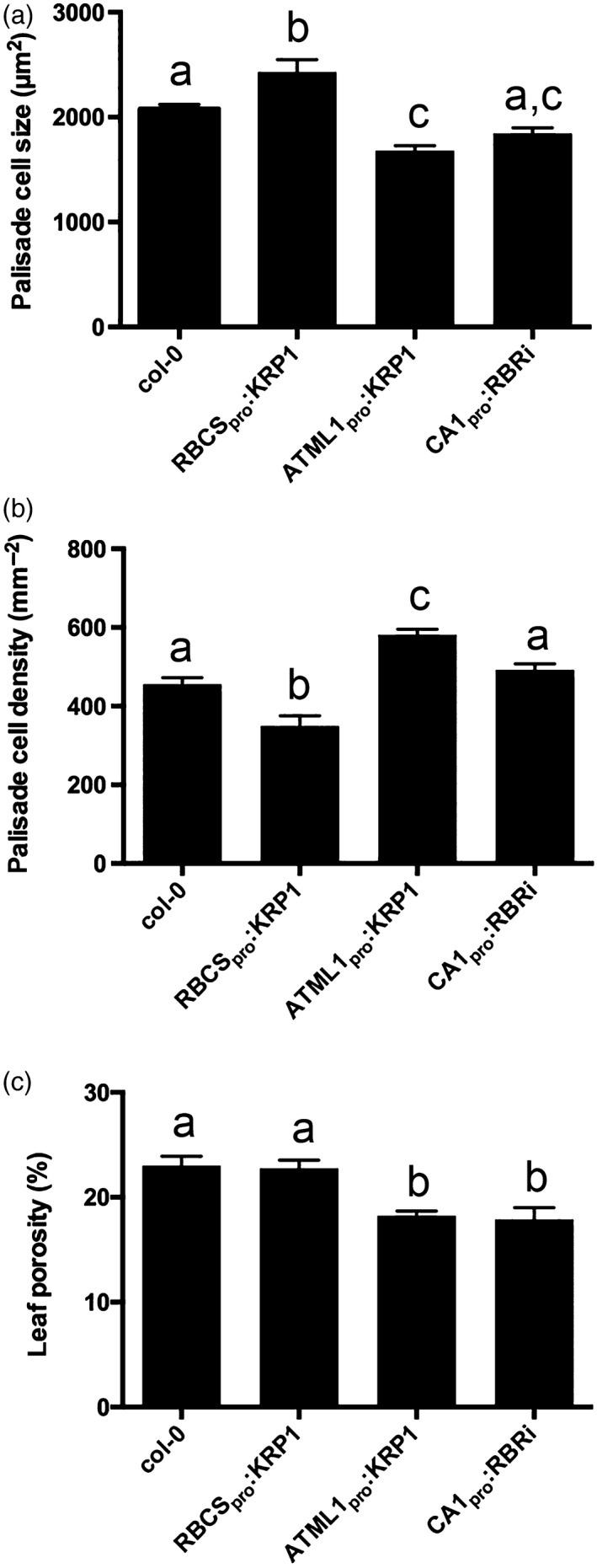
The modulation of cell cycle genes leads to changes in leaf cell size, density and porosity: (a) palisade cell size; (b) palisade cell density; and (c) leaf porosity in Col‐0, RBCS_pro_:KRP1, ATML1_pro_:KRP1 and CA1_pro_:RBRi leaves, as indicated. Values are means; error bars are SEMs. For (a) and (b) at least 15 cells were imaged per sample, with a total of 12 samples being analysed from three plants (*n *= 12); for (c), porosity values were measured in leaf discs taken from six independent plants (*n* = 6). Samples were compared with anova followed by *post‐hoc* Tukey's test. Columns indicated by identical letters within each analysis cannot be distinguished from each other at the 0.05 confidence limit.

**Figure 2:**
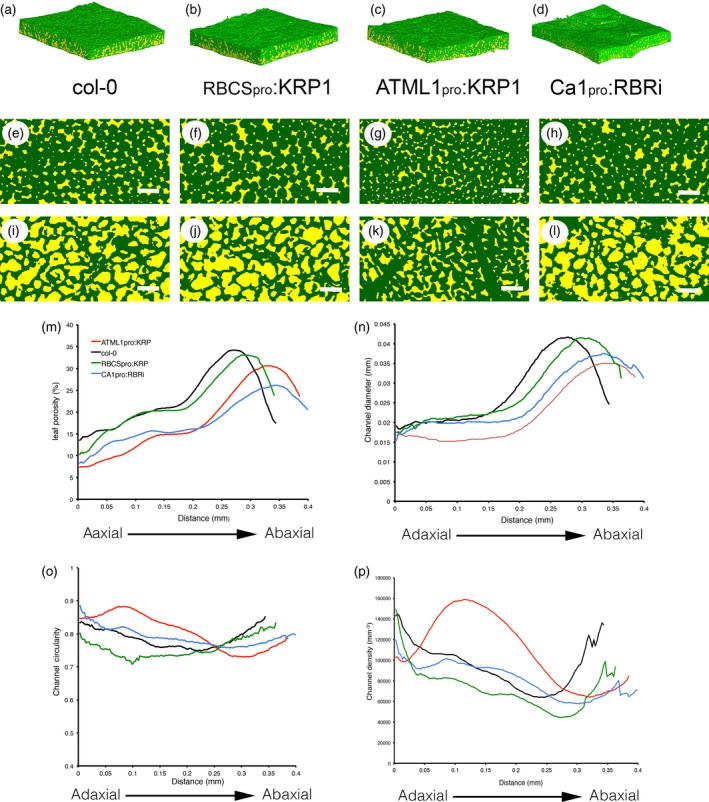
MicroCT imaging reveals variation in leaf airspace patterns. (a–d) 3D rendering of tissue blocks from (a) Col‐0, (b) RBCS_pro_:KRP1, (c) ATML1_pro_:KRP1 and (d) CA1_pro_:RBRi leaf samples. (e–l) Paradermal sections through the tissue blocks shown in a–d at a position towards the adaxial palisade surface (E–H) or towards the abaxial spongy surface (i–l). In (a–l), solid tissue has been painted green, and airspace has been painted yellow. (m) porosity values, (n) air channel diameter, (o) air channel circularity and (p) air channel density at distances below the adaxial surface of samples from wild‐type (WT, black), RBCS_pro_:KRP1 (green), ATML1_pro_:KRP1 (red) and CA1_pro_:RBRi (blue) leaves. For clarity, only mean values are indicated. Resolution in (e–l): 2.75 μm. Scale bars: 0.5 mm.

To investigate the consequences of cell division inhibition on leaf mesophyll architecture we created transgenic plants in which the *KRP1* gene was expressed under the control of an *RBCS* promoter sequence (RBCS_pro_:KRP1; see Experimental Procedures). Our analysis focused on leaf 8 at maturity. As expected, there was an increase in mean mesophyll cell size and a decrease in cell density, compared with Col‐0 (WT) leaves (Figure 1a,b). To quantify the outcome on the volume and pattern of airspace, samples were subjected to microCT analysis. A 3D rendering of a portion of a WT leaf is shown in Figure 2a, with paradermal sections through regions equivalent to the palisade and spongy layers shown in Figure 2e,i. These can be compared with equivalent images of RBCS_pro_:KRP1 leaves in Figure 2b,f,j, which suggest an increased cell size and altered airspace distribution in the transgenic plants. The data sets were analysed to compare quantitative values for porosity, air channel diameter, circularity and density at different planes within the sample from the adaxial to the abaxial surfaces (Figure 2m–p). These data showed that mean porosity (relative volume of airspace to total tissue volume) of Col‐0 WT tissue was generally low (15–20%) in the upper, adaxial part of the leaf (equivalent to the palisade region), but rose to a peak (30–35%) in the lower part of the leaf, the spongy tissue (Figure 2m). When the mean air channel diameter was measured, a similar distribution as seen for porosity was observed (Figure 2n), indicating that channels in the spongy region tended to be larger than those in the palisade layer. When channel circularity was considered, there was a minimum value towards the central part of the leaf (Figure 2o), and air channel density in WT leaves also declined in value across the palisade layer from the adaxial surface, reaching a minimum in the spongy region before rising towards the abaxial surface (Figure 2p).

The RBCS_pro_:KRP1 samples displayed a very similar pattern in all four parameters to that of the WT, but with the absolute values shifted. Thus, channel density was lower than in the WT in virtually all regions of the leaf (Figure 2p), but channel diameter was similar to that in the WT, with a slight shift in peak position reflecting the generally thicker mesophyll in these leaves (Figures 2n, S1b). There was a shift to lower mean circularity values in the RBCS_pro_:KRP1 samples (Figure 2o), indicating a more varied shape of the air channels in most portions of the leaf. Despite these shifts in channel density and shape, the overall level and distribution of porosity in the RBCS_pro_:KRP1 leaves was only slightly lower than that of the WT (Figures 1c, 2M).

In addition to targeting *KRP1* expression to the mesophyll, we also expressed the gene behind the *ATML1* promoter, leading to overexpression in the epidermis (see Experimental Procedures) and, as expected, an increase in epidermal cell size (Figure S1a). This manipulation also led to a significant decrease in mesophyll cell size and to an increase in mesophyll cell density (Figure 1a,b), substantiating previous observations (Bemis and Torii, 2007) and providing a set of transgenic plants displaying an inverse pattern of mesophyll cell size and density change to that observed in the RBCS_pro_:KRP1 leaves. MicroCT imaging of the ATLM1_pro_:KRP1 leaves suggested a higher tissue density in both the palisade and spongy layers (Figure 2c,g,k), and quantitative analysis substantiated this impression. A much lower porosity level was measured throughout the leaf, as well as a generally smaller channel diameter (Figure 2m,n). Channel circularity was higher than in the WT in the palisade region, but lower in the spongy region, leading to an almost inverted pattern of channel circularity (Figure 2o). When channel density was measured the pattern observed in the ATLM1_pro_:KRP1 leaves was also distinct from that in the WT and RBCS_pro_:KRP1 samples, with a distinct peak occurring in the palisade region of the ATLM1_pro_:KRP1 samples that was not observed in the other transgenic lines or WT plants (Figure 2p).

We also targeted increased mesophyll cell division by expressing an RBR1 RNAi construct using the *CA1* promoter sequence (see Experimental Procedures). The suppression of RBR1 is expected to remove the barrier to cell division, leading to the accumulation of more but smaller cells in the mesophyll (Kuwabara *et al*., 2011). Analysis of the CA1_pro_:RBRi leaves indicated that mesophyll cell size was decreased relative to WT, with a concomitant increase in cell density, although these changes were not as great as the changes observed in the ATLM1_pro_:KRP1 leaves (Figure 1a,b). MicroCT analysis of the CA1_pro_:RBRi samples (Figure 2d,h,l) indicated a general decrease in porosity in the adaxial and spongy regions, and a decrease in mean channel diameter, coupled with slight shifts in channel circularity and density compared with WT samples, but with the overall patterns of these parameters being similar to those observed in WT leaves (Figure 2m–p).

### Increased mesophyll cell density leads to an increase in photosynthetic capacity

**Figure 3:**
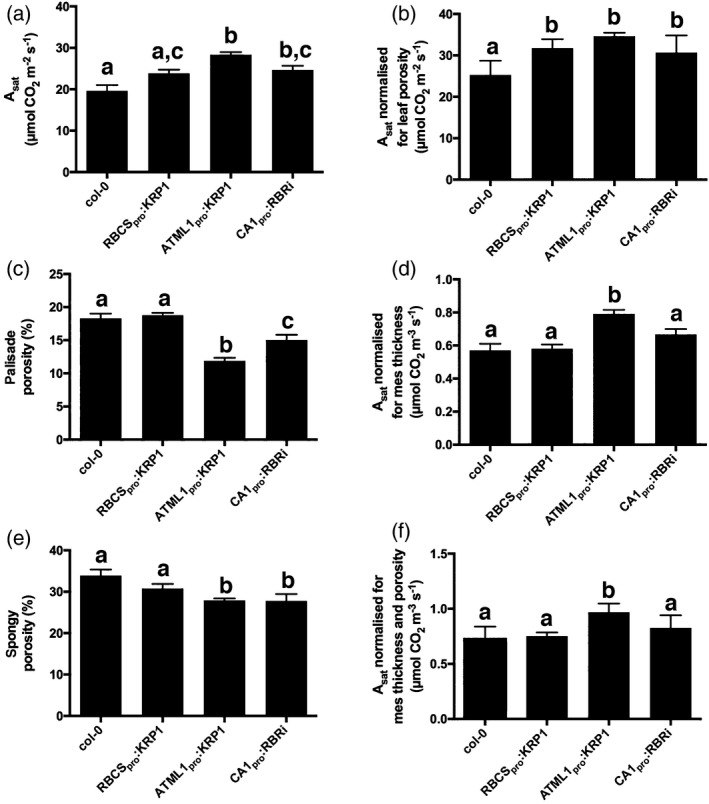
Modulation of cell cycle gene expression leads to altered CO_2_ assimilation rates. (a) Light saturated assimilation rate per area in Col‐0, RBCS_pro_:KRP1, ATML1_pro_:KRP1 and CA1_pro_:RBRi leaves, as indicated. (b) As in (A), but normalized for porosity. (c) Palisade mesophyll porosity for Col‐0, RBCS_pro_:KRP1, ATML1_pro_:KRP1 and CA1_pro_:RBRi leaves. (d) As in (a), but normalized for mesophyll thickness. (e) Spongy mesophyll porosity for the leaf samples as indicated in (d). (f) As in (a), but normalized for porosity and mesophyll thickness. Values are means, error bars indicate SEMs. For (a, b, d, f), values were measured in leaves from six independent plants (*n* = 6), except for Col‐0 (*n* = 5). For (c, d) *n* = 6. Samples were compared with anova followed by *post‐hoc* Tukey's test. Columns indicated by identical letters within each analysis cannot be distinguished from each other at the 0.05 confidence limit.

To investigate whether the altered patterns of cellular architecture described above were linked to any change in physiological performance, we performed a combined fluorescence/gas exchange analysis on the same leaf samples used for microCT. These data reveal a tendency for higher *A*_sat_ (maximum assimilation rate under saturating CO_2_) values in all three transgenic lines relative to the WT, with significant increases (*P* < 0.05) in the CA1_pro_:RBRi and ATLM1_pro_:KRP1 lines (Figure 3a). The pattern of decrease in porosity in the palisade layers (Figure 3c) generally mirrored the pattern of *A*_sat_ increase, suggesting that there might be a causal link, with the link to porosity of the spongy layers (Figure 3e) being less obvious. To investigate the potential underpinning mechanism for these observations we performed a number of further analyses.

Firstly, *A*_sat_ is measured on a per area basis, thus an increase in *A*_sat_ might reflect an increase in photosynthetic capacity per solid tissue volume or an increase in leaf thickness (with no change in photosynthetic capacity per tissue volume), or a combination of these changes. When the data shown in Figure 3a were normalized for porosity, a similar pattern of assimilation rates was still observed (Figure 3b), suggesting that the increased tissue density resulting from altered cell division pattern did not account for all of the observed increases in *A*_sat_. In contrast, normalization for differing leaf thickness led to assimilation rates in the RBS_pro_:KRP1 and CA1_pro_:RBRi lines similar to those calculated for WT samples (Figure 3d); however, when the different lines were compared for assimilation rate after normalization for both leaf thickness and mesophyll porosity, the ATML1_pro_:KRP1 samples still showed a significantly higher rate of CO_2_ assimilation, whereas the other two lines could not be distinguished from the WT (Figure 3f).

**Figure 4:**
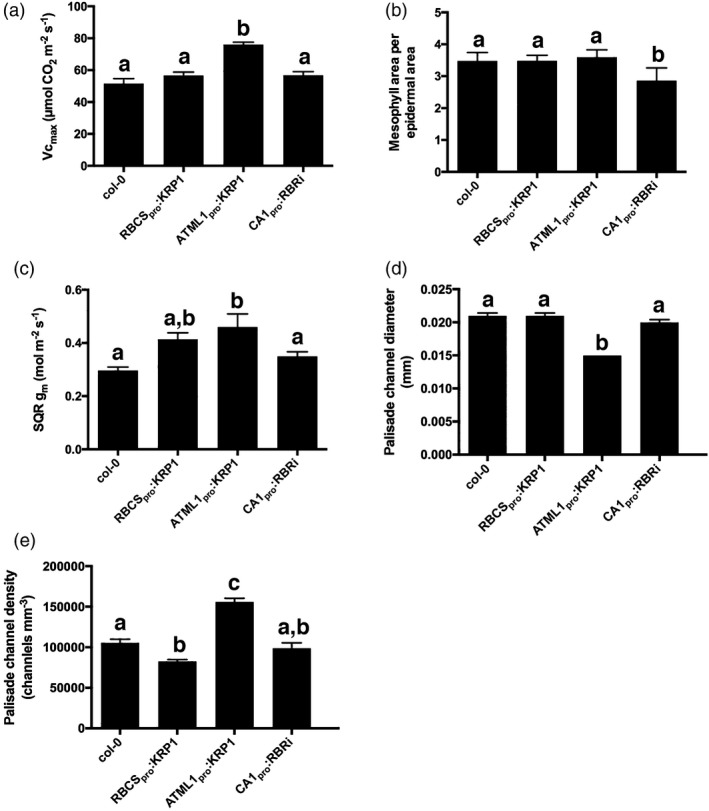
Evidence that the increased carboxylation rate is linked to decreased air channel diameter and increased air channel density: (a) *V*_cmax_; (b) exposed mesophyll area per one‐sided epidermal area; (c) mesophyll conductance, *g*_m_, after square‐root transformation to equalize standard deviation; (d) palisade channel diameter; (e) palisade channel density in Col‐0, RBCS_pro_:KRP1, ATML1_pro_:KRP1 and CA1_pro_:RBRi leaves, as indicated. Values are means; error bars are SEMs. Values were measured in leaves from at least five independent plants (*n* ≥ 5) except for: (a) Col‐0, *n* = 5; (c) Col‐0, *n* = 3). Samples were compared with anova followed by *post‐hoc* Tukey's test. Columns indicated by similar letters within each analysis cannot be distinguished from each other at the 0.05 confidence limit.

One possibility for the higher assimilation rate per solid leaf volume in the ATML1_pro_:KRP1 line was that there had been an upregulation of photosynthetic capacity. To investigate this possibility we first assayed the ability of the tissue to absorb and use light by directly measuring light absorptance (Figure S2a), assessing the fraction of light energy directed to photosystem II (PSII), αβ (Figure S2c), the maximum efficiency of PSII, *F*_v_/*F*_m_ (Figure S2e) and the initial/maximum quantum yield for CO_2_ fixation, *Y*(CO_2_)_LL_ (Figure S2g). These assays indicated no difference between any of the lines. The measurement of total pigment levels in the leaves indicated a significantly higher concentration in the ATML1_pro_:KRP1 leaves (Figure S2f), but the measurement of total leaf protein did not indicate any major increase in the ATML1_pro_:KRP1 samples, suggesting that the total quantity of Rubisco was not altered in a major way (Figure S2h). Calculation of *V*_cmax,_ which is a measure of the maximal Rubisco activity *in vivo* (von Caemmerer and Farquhar, 1981), however, indicated that the ATML1_pro_:KRP1 tissue had a significantly increased *V*_cmax_ (Figures 4a, S4c), consistent with the measured increase in *A*_sat_ and *CE* (Figure S2b). Overall, this evidence suggests that the measured increase in *A*_sat_ was not a reflection of an increased maximum efficiency of the photochemical machinery or level of Rubisco, but rather an increase in the proportion of light energy use through a reduced quenching of PSII in operational conditions, increased operational electron transport rate, *J* (Figure S2d), and an increased Rubisco activation state.

### Analysis of mesophyll conductance and airspace pattern

The rate of carbon fixation can also be influenced by the supply of CO_2_, and we therefore performed a series of analyses to test this hypothesis. Consistent with this proposal, there was a higher mesophyll conductance (*g*_m_) in the ATML1_pro_:KRP1 leaves than in the WT (Figure 4c), indicating a lower resistance to CO_2_ flux from the leaf airspaces to the active sites of Rubisco CO_2_ fixation within the chloroplasts. This leads to an ameliorated ratio between Rubisco carboxylation and photorespiration, allowing higher net assimilation for a given level of electron transport. The exposed mesophyll surface area (*S*_m_) is expected to play an important role in determining *g*_m_, as gas diffusion into and out of cells in the leaf must occur via the cell surfaces (Terashima *et al*., 2011), and is significantly slower in water than in air. Analysis of *S*_m_ did not reveal any significant difference between the different lines, however, except in the CA1_pro_:RBRi samples, which displayed a lower value of *S*_m_ (Figure 4b). Mesophyll conductance can also be influenced by physical parameters such as cell wall thickness and the arrangement and/or proximity of the chloroplasts to the plasma membrane of mesophyll cells; however, analysis of these parameters did not reveal any overt change that could account for the higher *g*_m_ in the ATML1_pro_:KRP1 leaves (Figure S3). For example, mesophyll walls in the ATML1_pro_:KRP1 cells had an average thickness of 0.164 ± 0.03 μm, whereas WT mesophyll cells had a average thickness of 0.096 ± 0.008 μm. Thus the ATML1_pro_:KRP1 mesophyll cell walls were actually thicker than those of the WT (Student's *t*‐test, *P *< 0.05, *n* > 4), which might, if anything, be expected to decrease CO_2_ conductance.

The air channel network in a leaf is involved both in the influx of CO_2_ and the efflux of water vapour, so that altered evapotranspiration might also influence the measured rate of CO_2_ assimilation (Terashima *et al*., 2011). Although the CA1_pro_:RBRi leaves had a higher stomatal density (Figure S1b), there were no significant differences between the other lines investigated, and the measured stomatal conductance did not significantly differ between any of the lines (Figure S1c), arguing against this having a major effect on *A*_sat_ or *V*_cmax_. An analysis of the relative roles of stomatal and non‐stomatal limitations on assimilation indicated that stomatal limitation increased in the ATML1_pro_:KRP1 leaves (Figure S4a,b), coinciding with the increase in *V*_cmax_ (normalized for both leaf thickness and porosity; Figure S4c). Overall, this evidence is consistent with the idea that greater *in vivo* Rubisco activity shifts the control of photosynthesis away from photosynthetic capacity and towards CO_2_ entry into the leaves.

As our microCT analysis had revealed that the ATML1_pro_:KRP1 leaves showed a distinct change in the pattern of air channel density, with the most extreme change being the decrease in channel diameter (Figure 2n,p), we took a closer look at these structural parameters across all four of the lines used. Consideration of palisade channel diameter (Figure 4d) and mean palisade channel density (Figure 4e) suggested some degree of correlation of these structural parameters with the measured physiological parameters of *A*_sat_ and *V*_cmax_. A correlation analysis of the various structural parameters and leaf performance measures reported in this paper indicated a number of potential relationships; however, most of these were not significant at the 0.05 confidence level. The exceptions to this were an inverse relationship of *A*_sat_ and spongy mesophyll porosity (*r* ^2^ = 0.82, *P* = 0.03) and *A*_sat_ and mean air channel diameter (*r* ^2^ = 0.78, *P *= 0.047). Most striking was the correlation between palisade air channel diameter and *V*_cmax_ (*r* ^2^ = 0.97, *P* = 0.019), supporting a tight linkage of these traits.

**Figure 5:**
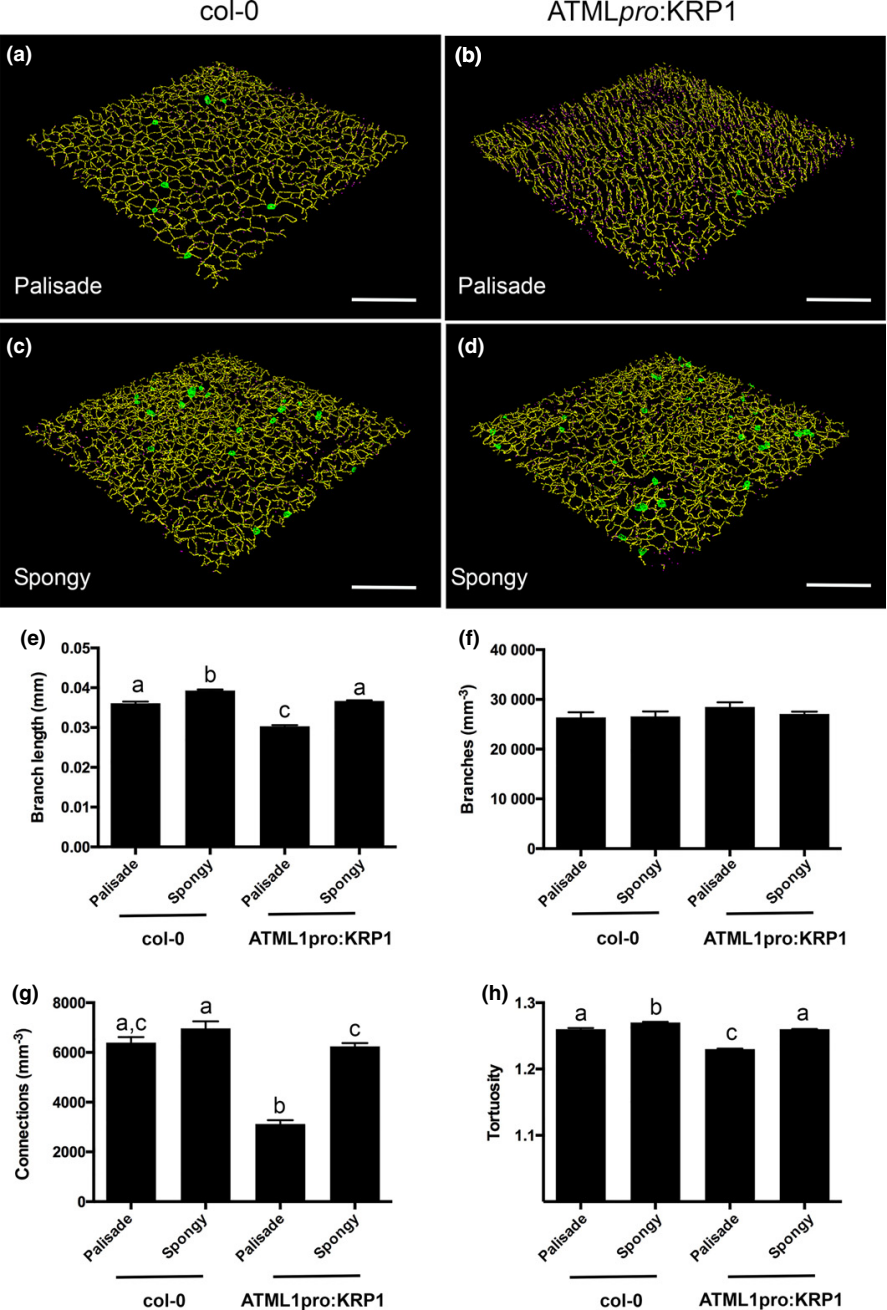
Analysis of ATML1_pro_:KRP1 leaves reveals an altered air channel network. (a, b) Images of the skeletonized air channel network in the palisade layer of Col‐0 (A) and ATML1_pro_:KRP1 (b) leaves. (c, d) As in (a, b), but images are of the spongy layer in the respective genotypes, as indicated. The voxels are colour‐coded: yellow, two connections per voxel; green more than two connections per voxel; magenta fewer than two connections per voxel (= network end point). (e) Mean branch length, (f) branch density, (g) connection density and (h) tortuosity values for the palisade and spongy mesophyll layers from Col‐0 and ATML1_pro_:KRP1 leaves, as indicated. Values are means; error bars are SEMs. Values were measured in leaves from six independent plants for Col‐0 and twelve independent plants for ATML1_pro_:KRP1. Samples were compared with anova followed by *post‐hoc* Tukey's test. Columns indicated by similar letters within each analysis cannot be distinguished from each other at the 0.001 confidence limit (except for g, WT spongy versus ATML1_pro_:KRP1 spongy, *P* < 0.01).

A deeper analysis of the air channel networks revealed further structural differences between the leaves that might influence photosynthetic performance. Representative images of the skeletonized palisade and mesophyll layers from control and ATML1_pro_:KRP1 leaves are shown in Figure 5a–d. The networks are colour coded so that green indicates regions where there are more than two connections per voxel and magenta indicates regions where there are fewer than two connections per voxel (equivalent to end points in the network). The palisade layer of the ATML1_pro_:KRP1 leaves was characterized by having fewer visible regions of high connectivity and an apparently higher density of network end points (magenta colour; compare Figure 5a and b). Quantitative analysis of the air space networks (Figure 5e–h) showed that the palisade layer of the ATML1_pro_:KRP1 leaves was characterized by a decrease in mean branch length and a network in which there were markedly fewer branch connections per leaf volume, i.e. network connectivity was significantly decreased (Figure 5g). As a consequence, the ATML1_pro_:KRP1 palisade layer air channel network displayed a significantly lower tortuosity (a measure of the twistedness of branches per volume) than the WT palisade tissue (Figure 5h).

## Discussion

### The impact of altered cell division patterns on leaf airspace volume and distribution

In this investigation we used targeted expression of cell cycle genes to alter cell division patterns in the leaf mesophyll, with the aim of investigating the outcome on airspace patterning. As expected, the expression of *KRP1* in the mesophyll led to the formation of fewer but larger cells, whereas directing *KRP1* expression to the epidermis led both to an increase in epidermal cell size and a decrease in mesophyll cell size (Verkest *et al*., 2005a,b; Weinl *et al*., 2005; Kuwabara *et al*., 2011). Previous publications have shown that *KRP1* fusion proteins have the ability to move between cells and that the outcome of *KRP1* on the cell cycle may be both level and context dependent, leading to the proposal that low levels of KRP1 may act to license entry to the G1 phase of the cell cycle (Verkest *et al*., 2005a,b). Although the precise mechanism by which the epidermal‐directed expression of KRP1 leads to an effect on sub‐epidermal cell size remains to be elucidated (Bemis and Torii, 2007), our data show that it can be reliably used as a tool to modify mesophyll cell division. Similarly, the observed decrease in palisade cell size and increase in cell density in the *CA1*_*pro*_ *:RBRi* transgenic leaves is consistent with the established role of this protein in suppressing cell proliferation (Borghi *et al*., 2010; Gutzat *et al*., 2012). Thus, by targeting the expression of well‐characterized cell cycle genes to different parts of the leaf we were able to generate plants with a spectrum of mesophyll cell sizes and densities, and consequently cell packing.

Our data clearly indicate that altering the cell division pattern has a knock‐on effect on the pattern and extent of air‐space formation in the leaf, as well as cell size. The mechanism by which this happens is essentially unknown. An array of cell wall enzymes are required for the controlled breakdown of cell wall components to allow for cell separation (McCann and Carpita, 2008). The simple observation that, for example, eudicot leaves generally form distinct palisade and spongy layers indicates that there is strong genetically programmed control of these processes. At the same time, it is clear that environmental influences modulate the extent of these cell separation processes, and one can envisage complicated feedback systems whereby altered cell division patterns lead to different physiological constraints within a tissue that might feed into cell size and separation to ensure an appropriate balance of, e.g. surface area for gas/vapour diffusion and photosynthesis/transpiration. Unravelling the molecular mechanism by which these cell wall changes are affected and integrated at a local and organ level with cell growth and division remains a major challenge for future research.

The use of microCT allowed us to quantify the outcome of these altered cell division patterns on 3D airspace patterning. The results showed that the modulation of leaf cell size and density does lead to modulation of the intervening airspace, but that the outcome is dependent on the manipulation performed. Thus, the promotion of smaller cells in the mesophyll was associated with an increase in channel density and a decrease in channel diameter, with a concomitant decrease in overall leaf porosity, i.e. a denser mesophyll was generated. Although the repression of mesophyll cell division led to an increase in cell size and a decrease in channel density, the concomitant changes in channel diameter and shape meant that the overall change in leaf porosity was very limited. The different outcomes on air channel volume and patterning of the different manipulations suggests that these are not simple scaling outcomes related to altered cell size, but rather indicate that there are feedback mechanisms linking cell size/shape and airspace within a leaf. The molecular mechanisms for these feedback processes remain obscure, but add to a picture (demonstrated, for example, by the phenomenon of compensation in leaves) in which size is somehow measured at a global scale so that the local scale is adjusted accordingly to produce a functional organ (Hisanaga *et al*., 2015). Whether physiological or biochemical activities act as part of the spatial mechanism of growth/function integration is open to speculation.

The use of 3D imaging allowed us to quantify the distribution of airspace across the leaf adaxial/abaxial axis. As expected, WT leaves displayed the classically described pattern in which relatively small channels at high density characterized the adaxial palisade region, whereas the abaxial spongy region contained relatively large channels at lower density. In the transgenics in which cell division was manipulated, this pattern was generally shifted up or down relative to the WT, suggesting that altered cell proliferation was modulating the developmental pattern rather than obliterating or radically changing it. This fits with our understanding of the adaxial and abaxial leaf domains being set early in development via a complex hierarchy of transcription factors and signalling events (Braybrook and Kuhlemeier, 2010). Although cell cycle genes must be part of the downstream effector network by which these patterns are translated into palisade and spongy differentiation, altered cell division alone does not generally appear sufficient to mask this initial pattern; however, there was an exception to this rule. In the ATML1_pro_:KRP1 plants (which showed the greatest decrease in cell size and increase in cell density) there was an abnormal and marked peak of channel density in the palisade. As far as we are aware, such a shift in airspace pattern has not been previously reported. These results show, firstly, that air channel pattern can be significantly manipulated within a leaf via the modulation of the cell division pattern. Secondly, as discussed below, such shifts in airspace channel pattern and size may underpin significant changes in photosynthetic performance.

### Altered cell division pattern, airspace and photosynthetic performance

Both an increase in mesophyll cell size and a decrease in mesophyll cell size tended to increase the leaf photosynthetic rate (Figure 3a); however, the mechanisms underpinning these measured increases were distinct. With respect to increased cell size, although there was a slight decrease in spongy porosity (indicating some increase in tissue density), the majority of the increase in photosynthetic rate measured per leaf area could be accounted for by an increase in leaf thickness. The simplest interpretation is that the increase in mesophyll cell size led to an increase in the depth of photosynthetic tissue in which gas exchange was measured, with little outcome of altered cell packing on photosynthetic performance. With respect to the leaves in which mesophyll cell size was decreased, the situation was more complicated. Neither increased leaf thickness nor the increased tissue density resulting from these manipulations accounted for all of the measured increase in photosynthetic rate in the leaves with the most extreme cellular phenotype. Assays of photosynthetic biochemistry did not suggest any major shift in either the ability of the tissue to absorb light energy or the capacity to transfer this energy into carbon fixation; however, there was evidence of a significant change in the *in vivo* activity of the carbon‐fixing enzyme Rubisco that underpinned the observed improvement in photosynthetic performance in this line. An additional possibility is that the structural changes resulting from the generation of a mesophyll containing such small cells somehow led to an increased CO_2_ conductance within the leaf.

Mesophyll conductance (*g*_m_) is a complex trait that has been postulated to play an important role in photosynthetic efficiency (Flexas *et al*., 2008; Kaldenhoff, 2012; Adachi *et al*., 2013). Essentially, an improved flux of CO_2_ within the mesophyll should lead to a better supply of CO_2_ to Rubisco, and hence an improved rate of carbon fixation. In support of this hypothesis, *g*_m_ was higher in the ATML1_pro_:KRP1 line compared with both the WT and the other transgenic lines. Measurements of structural parameters that might influence *g*_m_ (exposed mesophyll surface area, chloroplast distribution within the mesophyll cells and mesophyll cell wall thickness) did not reveal any changes in the ATML1_pro_:KRP1 samples that might underpin the observed increase in *g*_m_. In contrast, distinct structural changes were observed in this line that can plausibly be traced to the manipulation performed. These included a high palisade air channel density, a significant decrease in air channel diameter, and a decrease in air channel connectivity and tortuosity. Predicting the outcome of such altered air channel patterns and dimensions on diffusion within a leaf and gas exchange with the surrounding tissue is not trivial, and requires various assumptions on, for example, laminar gas flow, distribution of CO_2_ sinks within the leaf, as well as estimations of the counter flow of water vapour within the same channels (Parkhurst, 1994). Nevertheless, the altered structural parameters of the airspace network in the palisade of the ATML1_pro_:KRP1 leaves are consistent with a network in which CO_2_ is expected to permeate more rapidly and to a higher resolution, thus potentially decreasing the pathway length for CO_2_ diffusion in the liquid phase. Such changes would contribute to the observed increase in *g*_m_ and increased maximum assimilation rate. A greater understanding and modelling of the local concentrations and flux of CO_2_ and water vapour within the mesophyll air channel networks will be required to test this hypothesis.

In conclusion, the results reported here indicate that altered cell division patterns in the leaf lead to altered patterns of airspace, and can lead to an increase in the photosynthetic rate. Although some of this increase can be related to changes in gross leaf morphology (thicker leaves) and internal cellular architecture (less porous, denser leaves containing smaller cells), leaves with more extreme increases in cell density appear to have other factors at play, namely increased Rubisco activity and higher *g*_m_. We propose that the generation of a higher density network of shorter, smaller diameter but less connected air channels in the palisade layer plays a role in the measured increase in *g*_m_. Our data indicate that a complex interaction of cell division patterning, growth and separation informs airspace patterning, with the integration of these factors having a major influence on leaf photosynthetic performance. The molecular mechanisms coordinating these cellular and organ‐level processes await elucidation.

## Experimental Procedures

### Plant growth

Seeds were stratified on wetted filter paper at 4°C in the dark for 7 days, then germinated on M3 compost in 4 × 4 × 6‐cm brown plastic pots. All plants were grown in a Conviron MTPS 120 growth room (http://www.conviron.com), with a 12‐h photoperiod set to 21/15°C day/night temperatures, 60% relative humidity (RH) and 200 μmol m^−2^ sec^−1^ photosynthetic photon flux density (PPFD) at canopy height. Only the eighth leaves of replicate plants were subjected to absorption measurements, pigment content quantification or combined fluorescence/gas exchange and subsequent microCT analysis, as described below. For all measurements, leaf 8 was sampled between 34 and 38 days after sowing, a time by which leaf 8 had reached its final size but had not undergone any obvious signs of senescence. For Dex induction, 10 μl of 10 μm Dex/DMSO solution was applied to the shoot apex on days 15, 16 and 17 when leaf 8 was initiated. Forty‐eight hours after the final Dex application, a leaf from at least three representative plants each was harvested for GUS assay.

For the selection of transgenic lines, seeds were surface sterilized, stratified at 4°C in the dark for 7 days, and then grown for selection on half‐strength Murashige and Skoog salt mix (Sigma‐Aldrich, https://www.sigmaaldrich.com), 1% (w/v) sucrose and 0.8% (w/v) plant agar (Duchefa Biochemie, https://www.duchefa-biochemie.com), with appropriate antibiotic selection (20 μg ml^−1^ hygromycin or 50 μg ml^−1^ kanamycin). Plates were kept in a growth chamber (Snijders Scientific, now Snijders Labs, http://www.snijderslabs.com) with a 16‐h photoperiod at 100 μmol m^−2^ sec^−1^ PPFD and constant temperature of 22°C before either collection for analysis or transfer to M3 soil for seed production in a 16‐h photoperiod at 22°C constant temperature, 60% RH and 100 μmol m^−2^ sec^−1^ PPFD at canopy height.

### Creation of transgenics and mutant characterization

The transgenic line *RBCS*_*pro*_ *:KRP1* was made by introducing a previously described GUS.YFP.KRP1 sequence in the Gateway‐compatible pEN01 vector (Weinl *et al*., 2005) by gateway recombination into pRBC‐pAM‐PAT‐GW, where the *35S* promoter has been exchanged with the sub‐epidermal specific promoter RBCS2b_pro_ (Kim *et al*., 2005) using restriction site cloning. Targeting of KRP1 under the control of the *RBCS2b* promoter was expected to lead to the expression of the gene predominantly in the mesophyll. Localization of transgene expression to the mesophyll was confirmed via GUS histochemical analysis (Figure S5e), although some staining was also observed in the epidermis. For this line (and others described below), measurements of cell size and density were obtained across 12 images, each with approximately 15 cells, from at least three independent plants using imagej (https://imagej.nih.gov/ij/).

The transgenic line *ATML1*_*pro*_ *:KRP1* was constructed by introducing a previously described YFP.KRP1 sequence (Weinl *et al*., 2005) behind the ATML1_pro_ via Gateway recombination cloning. The construct was introduced using gateway recombination into the vector pATML1‐pAM‐PAT‐GW that has been previously described (Bouyer *et al*., 2008) and transformed into *Arabidopsis thaliana* (Col‐0) using a conventional floral‐dip method. Seeds were collected and, after selection, based on the antibiotic selection marker, used to generate homozygous T_3_ lines (confirmed by genotyping) that were used for experiments. Epidermal expression was confirmed by confocal microscopy using the YFP tag included in this construct (Figure S5h). This revealed relatively large epidermal cells, confirmed by measurements (Figure S1a).

The line CA1_pro_:RBRi was made by first introducing a 2124‐bp fragment of the PPCA1 promoter from *Flaveria trinervia* (Gowik *et al*., 2004) into a Dex‐inducible/LhGR activator construct pBin‐LR‐LhGR^2^, which also contains the GUS reporter gene sequence (a gift from I. Moore) by gateway cloning. The promoter was amplified using primers: 5′‐CACCAAGGACTCACCAGGACAGG‐3′ and 5′‐TACTCACACCCTTGCTTAATAC‐3′, using pPPCA1‐pAM‐PAT GW as the template (Bouyer *et al*., 2008). In order to clone the RBRi sequence we used CATMA3a11240 GST [N264209, which contains a 169‐bp fragment of the RBR1 gene (AT3G12280); NASC, http://arabidopsis.info] to clone into pOpOff1 (RNAi construct, gift from I. Moore) by Gateway cloning. Both constructs, pBin‐LR‐LhGR^2^:pPPCA1 and pOpOff1:RRBi, were transformed independently into Col‐0 using a conventional floral‐dip method. Homozygous T_3_ lines were obtained for each construct and crossed together in order to obtain lines that expresses RBRi under the Dex‐inducible CA1 promoter. Homozygous CA1_pro_:RBRi lines were used for further analysis, with GUS histochemical analysis of induced tissue revealing that target gene expression was restricted to the mesophyll (Figure S5k). Although CA1_pro_:RBRi lines showed a decrease in palisade cell size (Figure 1a), this was observed in both induced and non‐induced lines and qPCR analysis did not indicate a significant reduction in transcript level after induction (two independent transgenic lines). In the experiments described for the analysis of leaf cellular architecture and physiology, data for induced and non‐induced lines were combined because they did not separate in subsequent analyses, but were distinct from Col‐0.

### GUS histochemistry

For analysis of GUS reporter gene expression, plants were submersed in X‐GlcA substrate (5‐bromo‐4‐chloro‐3‐indolyl β‐d‐glucuronic acid cyclohexylammonium salt, 0.5 mg ml^−1^) in 100 mm NaH_2_PO_4_ (pH 7.0) in the presence of potassium hexacyanoferrate II + III (1 mm) at 37°C overnight, after vacuum infiltration for 20 min. Samples were rinsed in 70% v/v ethanol and fixed in 100% EtOH:glacial acetic acid (7:1 v/v) at room temperature (19–22°C) overnight until the complete removal of chlorophyll. Samples were embedded for sectioning using Technovit 7100 (Kulzer, http://kulzer.com) following the manufacturer's instructions. Sections (8 μm) were taken using a Leica RM2145 microtome. After staining with 0.02% (w/v) Saffranin O, samples were observed under an Olympus BX51 light microscope (https://www.olympus-global.com).

### Gas exchange and fluorescence measurements

Fluorescence and gas exchange were simultaneously measured with a LI‐6400 XT equipped with a 2‐cm^2^ leaf chamber fluorometer (Li‐6400‐40; LI‐COR, https://www.licor.com). For all measurements, leaf temperature was maintained at 21°C and cuvette RH was controlled at ~60% to match growth conditions. In order to minimize diffusive limitations, light‐response curves were performed at a CO_2_ concentration in the measurement cuvette (*C*_a_) of 1000 μl L^−1^, under decreasing irradiance, from 1500 to 0 μmol m^−2^ sec^−1^ PPFD. *F*_v_/*F*_M_ was measured on dark‐adapted leaves at 400 μl L^−1^ *C*_a_. *A*/*C*_*i*_‐response curves were measured on six replicate plants per genotype at 1200 μmol m^−2^ sec^−1^ PPFD. Leaves were pre‐acclimated for about 20 min at *C*_a_ = 100 μl L^−1^ to induce stomatal opening. Once *g*_s_ had reached a threshold of 0.3 mol H_2_O m^−2^ s^−1^, *C*_a_ was set to 400 μl L^−1^ CO_2_ and acclimated until *A* and *g*_S_ were stable. *A*/*C*_i_ curves were measured at *C*_a_ values of 50, 100, 150, 200, 250, 300, 400, 500, 600, 800, 1000, 1300 and 1600 μl L^−1^ (Long and Bernacchi, 2003; Evans and Santiago, 2014). For each leaf measured, an *A*/*C*_i_ curve was first performed at ambient oxygen concentrations (i.e. approximately 21%), and then a second *A*/*C*_i_ curve with identical settings was performed with the cuvette atmosphere supplied with 2% O_2_. Steady‐state values of *g*_s_ were taken or calculated at a *C*_a_ value of 400 μl L^−1^ during the *A*/*C*_i_ curve measurements at ambient O_2_ concentration.

Light‐ and *A*/*C*_*i*_‐response curves were analysed following the method described by Bellasio *et al*. (2016). Briefly, the PPFD dependence of gross assimilation (*GA*) was described empirically by a non‐rectangular hyperbola, so as to estimate, by iterative fitting, the respiration in the light (*R*_*LIGHT*_), the maximum quantum yield for CO_2_ fixation [*Y*(*CO*_*2*_)_*LL*_] as the initial slope of the light‐response curve [*Y*(*CO*_*2*_)_LL_] and the light‐saturated *GA* (*GA*_SAT_) as the horizontal asymptote. The light compensation point (*LCP*) was estimated by solving the fitted light‐response curve for *GA *=* R*_LIGHT_. An empirical non‐rectangular hyperbola was fitted to the *A*/*C*_*i*_ curves under ambient and low O_2_ to estimate the maximal carboxylating efficiency (*CE*) as the initial slope, the *C*_*i*_/*A* and *C*_*i*_/*GA* compensation points (Γ and *C*_i_*, respectively), and the CO_2_‐saturated *A* (*A*_SAT_). The *in vivo* Rubisco specificity factor (*S*_*C/O*_) was estimated under the assumption that Γ* and *C*_i_*, from all‐plants‐averaged *C*_i_* (47.3 μmol mol^−1^; Brooks and Farquhar, 1985), and resulted in an *S*_*C/O*_ estimate of 2114 bar bar^−1^. Thus, these values were used in the curve‐fitting procedure for all genotypes. The fraction of *PPFD* harvested by PSII (a quantity called αβ) was derived by linear regression of *Y*(*II*) plotted against the quantum yield for CO_2_ fixation [Φ_CO2_, or *Y*(*CO*_*2*_) here] using data from the *A*/*C*_*i*_ curve measured under low O_2_ (Valentini *et al*., 1995). With αβ, *b* and measured values of *Y*(*II*)*,* the electron transport rate (*J*) was derived for each point of the *A*/*C*_*i*_ curve measured under ambient O_2_ (Valentini *et al*., 1995). Mesophyll conductance to CO_2_ diffusion (*g*_m_) was estimated using the NRH‐A variant of the method proposed by Yin and Struik (2009), using the lesser root to the equation A23 from von Caemmerer (2013), by the iterative fitting of *A* from the *A*/*C*_*i*_ curves with previously derived *J* and *R*_LIGHT_ values. The CO_2_ concentration at the site of Rubisco carboxylation (*C*_C_‐based Rubisco kinetic parameter *V*_CMAX_) was estimated by fitting the ‘full Farquhar model’, as developed by Ethier and Livingston (Ethier and Livingston, 2004), to the Rubisco‐limited part of the *A*/*C*_*i*_ curve, with Γ, *g*_m_, and *R*_LIGHT_ previously determined, and assuming a *K*_C_(1 + O/*K*_O_) of 560 μbar.

*A*_sat_ values were normalized for mesophyll thickness and/or porosity using paired data obtained from microCT of the same leaves used for gas‐exchange analysis (described below). These data are provided in Table S1.

### Absorptance and pigment analysis

Light absorptance was measured in the same central region of the leaf where both photosynthesis and microCT measurements were taken (Rackham and Wilson, 1968). Leaves were irradiated using two lasers: a blue diode‐pumped solid‐state laser (emission 468 and 472 nm; Laser 2000, http://www.laser2000.co.uk), and a red supercontinuum laser (emission 495 and 700 nm; NKT Photonics, http://www.nktphotonics.com/). Radiation was measured with a Taylor‐type integrating sphere (Labsphere Inc., https://www.labsphere.com) and a spectrometer with a 50‐μm slit, a diffraction grating blazed at 500 nm, and a spectral range between 300 and 720 nm (Oriel, https://www.newport.com/b/oriel-instruments). The total light absorptance of a leaf was then calculated as the average absorptance of all wavelengths measured across 468 and 472 nm and 495 and 700 nm. Photosynthetic pigments were quantified on four replicate plants per genotype. Two discs (8.5 mm in diameter) were punched from each side of the midrib, fresh weight measured and rapidly ground with 80% (v/v) acetone. Visible spectra were measured (400 and 700 nm) at 1‐nm steps and total pigment levels were quantified according to the method described by Lichtenthaler and Wellburn (1983).

### X‐ray microCT image acquisition and analysis

Leaf 8 of each plant was imaged using an X‐ray microCT scanner (Nanotom; General Electric, http://www.ge.com) in two steps: whole‐leaf imaging to investigate leaf morphological features and imaging of two leaf discs (5 mm in diameter). These discs were cut symmetrically from each side of the mid‐rib. Samples were kept static during image acquisition to maximize image quality. microCT scans of the whole leaves were obtained at a spatial resolution of 15 μm with an energy of 65 kV and 140 μA, collecting 720 projections with an exposure time of 750 ms, resulting in a total scan time of 18 min per leaf. Data sets of leaf discs were acquired at 2.75 μm of spatial resolution, using the same energy and exposure settings as for the whole leaf but collecting 1200 projections resulting in a total scan time of 30 min per leaf disc.

The 2D projections (radiographs) acquired during the scans were reconstructed (Datos|X; General Electric) into 3D volumes using a filtered back‐projection algorithm, rendered and converted into stacks of Tiff images (vg studio max 2.2; Volume Graphics, https://www.volumegraphics.com). Image stacks were used to create a mask separating the leaf or leaf discs from the background and any holding devices (avizo 6.0; ThermoFisher Scientific, https://www.fei.com). In the case of whole‐leaf data sets, the masks were directly quantified using the imagej plug‐in bonej (http://bonej.org). Image stacks with leaf discs separated from the background were cropped to remove any damaged area on the edges. Cropped and aligned image stacks of leaf discs were thresholded using imagej isodata and minimum algorithms to obtain stacks of images representing the leaf disc mask and plant material only. The generated image stacks were used as the input to the imagej image calculator, and the function XOR was used to generate image stacks with only pore space visible. As the last stage of image analysis, the structural descriptors of intercellular pore space, such as porosity, channel diameter, mesophyll surface area, connectivity, circularity and skeleton analysis, were obtained using the imagej particle analyser and bonej. 3D renderings of connected channel networks were generated using RGB stacks from imagej imported into vg studio max. For analysis we defined a region of interest between the boundary of the upper epidermis/mesophyll and lower epidermis/mesophyll. This allowed us to use mesophyll thickness (rather than leaf thickness) in our analysis of photosynthetic parameters. Mesophyll surface area was normalized to the one‐sided surface area of the leaf disc to calculate the ‘exposed mesophyll surface area’ parameter. Two subsets of the porosity, channel diameter and circularity data were taken along the thickness of each leaf, at distances of one‐quarter and three‐quarters from the adaxial surface to represent palisade and spongy mesophyll types, respectively. For network analysis, tortuosity was calculated as branch length/Euclidean distance, where Euclidean distance is the mean distance (mm) between the V1 and V2 vertices (end points) of each branch within a volume.

### Confocal and standard light microscopy

For light microscopy, leaves were dehydrated in an ethanol series, fixed in a 7:1 ethanol:glacial acetic acid solution, then imaged using differential interference contrast microscopy (Kuwabara *et al*., 2011, 2011; Figure S5a–d, f, g, i, j)**.** Cleared leaves were photographed using imaging software (cell b; Olympus) and a DP71 camera on an Olympus BX51. Confocal images from living leaves were collected using an Olympus FV1000 with SIM‐scanner on a BX61 upright microscope, with Olympus fluoview fv1000 software. Chlorophyll excitation was at 488 nm and emission was between 650 and 710 nm. YFP was excited at 515 nm and emission collected between 530 and 570 nm. Gain and offset levels were kept constant during all measurements. Three different plants were imaged per line and two leaf discs were collected per leaf from either side of the midrib then mounted in perfluorodecalin. Two areas were imaged in each leaf disc. A *z*‐stack was taken of the palisade mesophyll layer with a stepsize of 1 μm, and the clearest parts of the images of the chloroplasts were merged in Adobe photoshop cs2 to give representative images for analysis.

### Transmission electron microscopy

Samples were fixed in 3% (w/v) glutaraldehyde in 0.1 m sodium cacodylate buffer overnight, washed twice in the same buffer (15 min each) then post‐stained in 1% (w/v) osmium tetroxide for 1 h. After a brief rinse in water, samples were washed twice for 15 min in cacodylate buffer, then dehydrated through an aqueous ethanol series (50, 75, 80, 90, 95 and 100%), with 15 min for each wash. After two 15‐min washes in epoxypropane (EPP), samples were infiltrated in 50% (w/v) araldite resin in EPP overnight on a rotor. The solution was replaced with fresh araldite resin twice over 8 h on a rotor. Samples were then embedded in fresh araldite resin in coffin moulds and left at 60°C to cure for 2–3 days. Sections (85 nm) were then taken using a Leica UC6 and collected onto 400‐mesh Formvar copper grids. After staining in uranyl acetate and Reynold's lead citrate, sections were viewed on an FEI tecnai Biotwin TEM at 80 kV. Images were recorded on an Orius digital camera using Gatan digital micrograph (Gatan, http://www.gatan.com/). Samples were taken from the middle of the lamina on mature leaf 8 from at least four independent plants, and multiple images were taken from sections from each tissue block. For analysis of cell wall thickness, care was taken to ensure that sections were perpendicular to the wall, as indicated by the sharp boundaries within the image and minimal indication of shearing.

## Authors' Contributions

CL, RP, MRL, AM, JS, MB, AM, AD and DB performed the experiments; CL, RP, MRL, AM, JS, CB, DB, AS, CS, CPO, SR, SM and AJF analysed the data; CL, RP, MRL, AM, DB, AS, CS, SM and AJF designed the experiments; CL, RP and MRL led the organisation and management of different parts of the project; CL, RP, MRL, CB, AS, CS, CPO, SR, SM and AJF contributed to writing the manuscript; SM and AJF planned and led the project.

## Conflict of Interest

The authors have no conflict of interest.

## Supporting information


**Figure S1.** Epidermal cell size, stomatal density and stomatal conductance.

**Figure S2.** Analysis of light absorption and photosystem efficiency.

**Figure S3.** Analysis of chloroplast and cell structure.

**Figure S4.** Analysis of stomatal and non‐stomatal limitation.

**Figure S5.** Generation and characterization of transgenic Arabidopsis.

**Table S1.** Physiology and imaging data.
