# Supplementary material for: Cell density and airspace patterning in the leaf can be manipulated to increase leaf photosynthetic capacity
Source: Plant J. 2017 Nov 15;92(6):981–94. doi: 10.1111/tpj.13727 (PMC5725688; doi:10.1111/tpj.13727)
Supplement: Supplementary file 1 — Figure S1. Epidermal cell size, stomatal density and stomatal conductance. [file TPJ-92-981-s001.pdf]

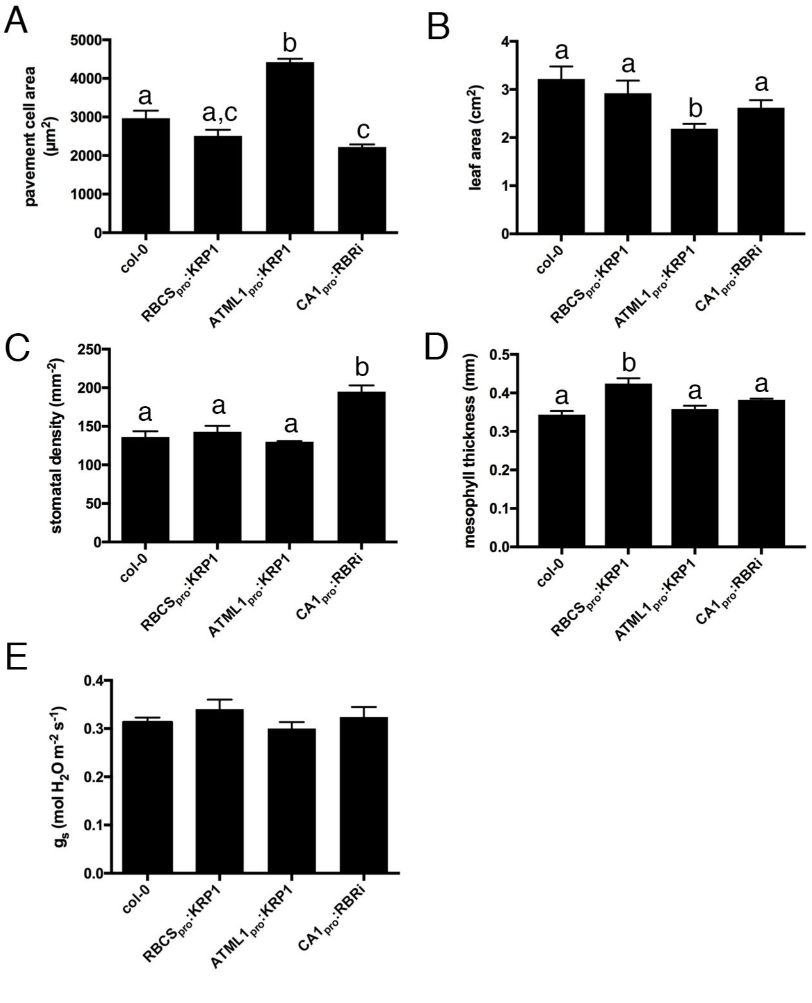

Sup Fig. 1

Epidermal cell size, stomatal density and stomatal conductance

(A) Upper epidermal pavement cell area **(B)** area of leaf 8 at maturity **(C)** stomatal density in upper epidermis **(D)** mesophyll thickness and **(E)** stomatal conductance, *g*s, in *Col-0*, $RBCS_{pro}$:KRP1, $ATML1_{pro}$:KRP1 and $CA1_{pro}$:RBRi leaves, as indicated. Values are means, error bars = sem. Values were measured in leaves from at least four (B) or at least five (A,C,D,E) independent plants. Samples were compared with ANOVA followed by a post-hoc Tukey test. Columns indicated by identical letters within each analysis cannot be distinguished from each other at the 0.05 confidence limit.
