# Supplementary material for: Cell density and airspace patterning in the leaf can be manipulated to increase leaf photosynthetic capacity
Source: Plant J. 2017 Nov 15;92(6):981–94. doi: 10.1111/tpj.13727 (PMC5725688; doi:10.1111/tpj.13727)
Supplement: Supplementary file 2 — Figure S2. Analysis of light absorption and photosystem efficiency. [file TPJ-92-981-s002.pdf]

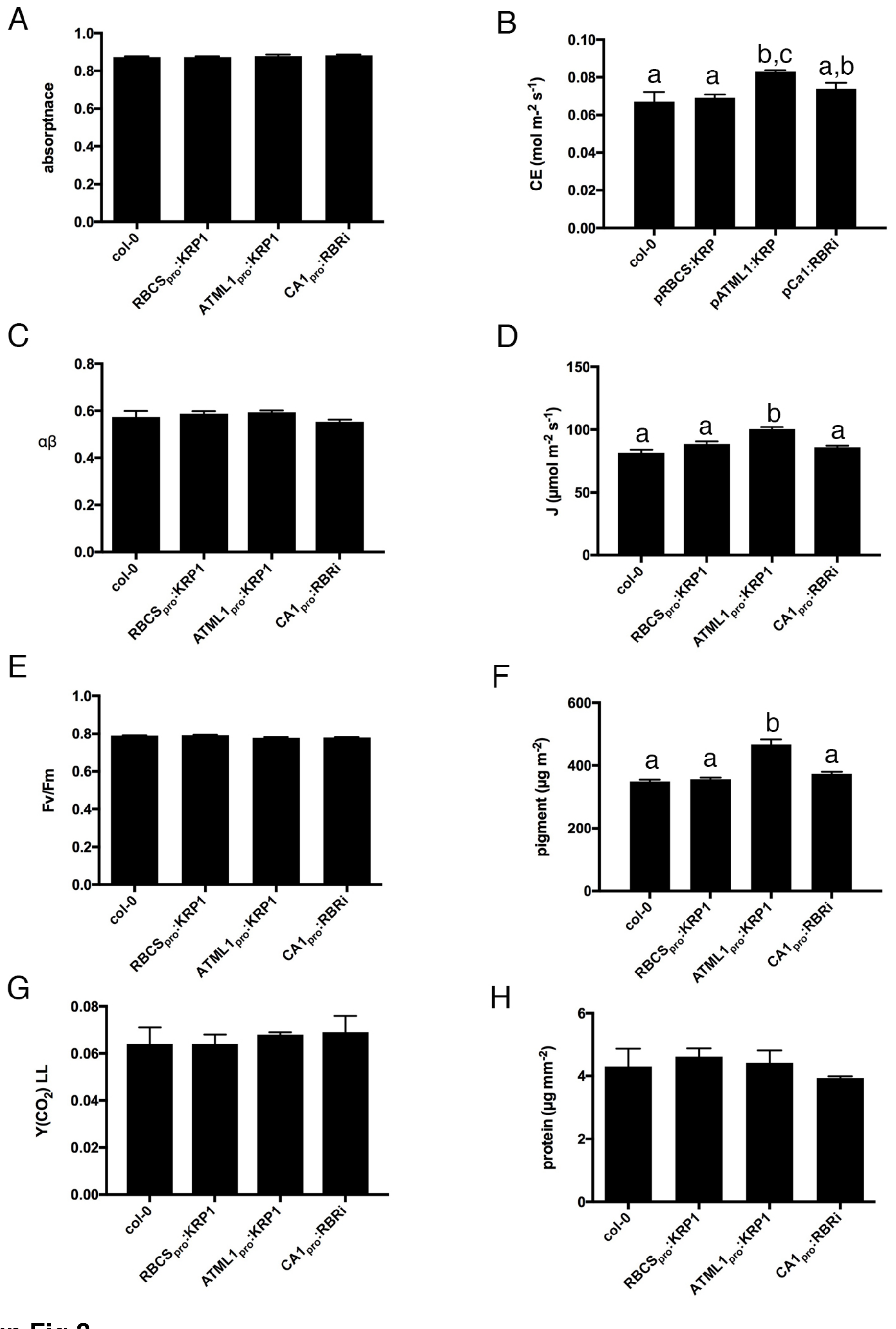

Sup Fig 2

Analysis of light absorption and photosystem efficiency

(A) Light absorptance **(B)** carboxylation efficiency, *CE* **(C)** the fraction of *PPFD* harvested by PSII, *αβ* **(D)** electron transport rate, J_{400} **(E)** the maximum efficiency of photosystem II, F_v/F_m **(F)** the total pigments per area **(G)** initial/maximum quantum yield for CO_2 fixation, $Y(CO2)_{LL}$ and **(H)** total protein concentration per area in *Col-0*, $RBCS_{pro}$:KRP1, $ATML1_{pro}$:KRP1 and $CA1_{pro}$:RBRi leaves, as indicated. Values are means, error bars = SEM. For A, B, D, F, G n ≥3; C ≥ 5; E ≥ 6. Samples were compared with ANOVA, followed by a post-hoc Tukey test. Columns indicated by identical letters within each analysis cannot be distinguished from each other at the 0.05 confidence limit.
