# Supplementary material for: Cell density and airspace patterning in the leaf can be manipulated to increase leaf photosynthetic capacity
Source: Plant J. 2017 Nov 15;92(6):981–94. doi: 10.1111/tpj.13727 (PMC5725688; doi:10.1111/tpj.13727)
Supplement: Supplementary file 3 — Figure S3. Analysis of chloroplast and cell structure. [file TPJ-92-981-s003.pdf]

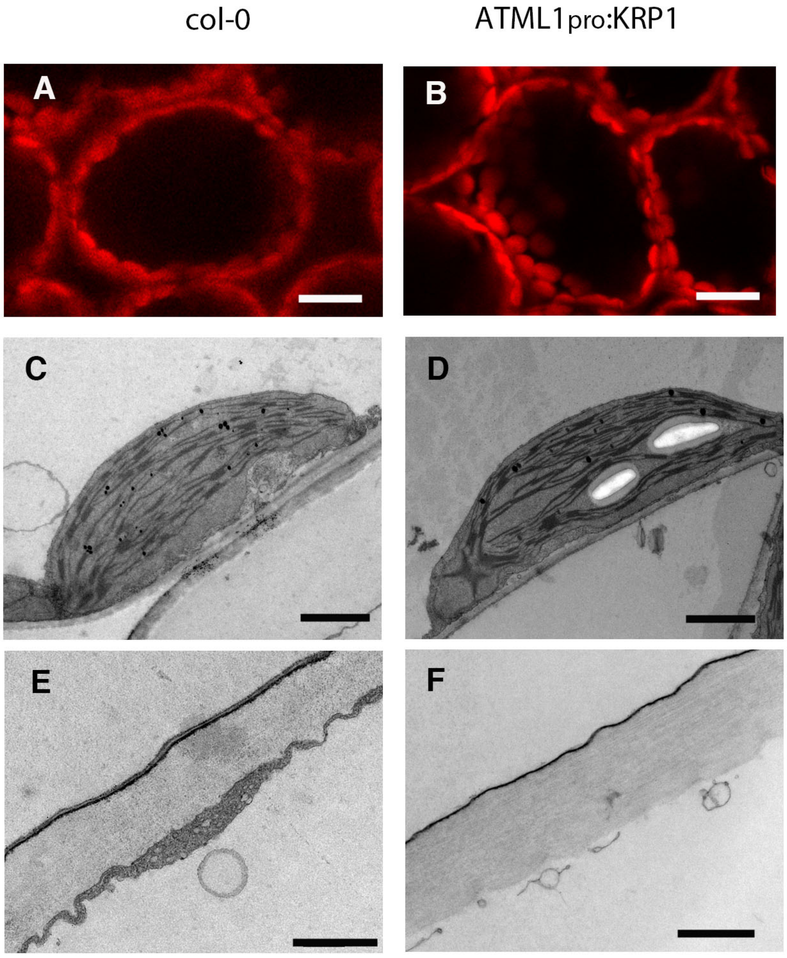

Sup Fig. 3

Analysis of chloroplast and cell structure

(A,B) Confocal images of palisade cells in (A) col-0 and (B) *ATML1pro:KRP1* leaves revealing chlorophyll autofluorescence in plastids (red).

(C,D) TEM images of a chloroplast from a palisade cell in (C) col-0 and (D) *ATML1pro:KRP1*.

(E,F) TEM images of cell wall from a palisade cell in (E) col-0 and (F) *ATML1pro:KRP1*.

Scale bar: A,B = 50 µm; C,D = 2 µm; E,F = 0.5 µm
