# Supplementary material for: Cell density and airspace patterning in the leaf can be manipulated to increase leaf photosynthetic capacity
Source: Plant J. 2017 Nov 15;92(6):981–94. doi: 10.1111/tpj.13727 (PMC5725688; doi:10.1111/tpj.13727)
Supplement: Supplementary file 4 — Figure S4. Analysis of stomatal and non‐stomatal limitation. [file TPJ-92-981-s004.pdf]

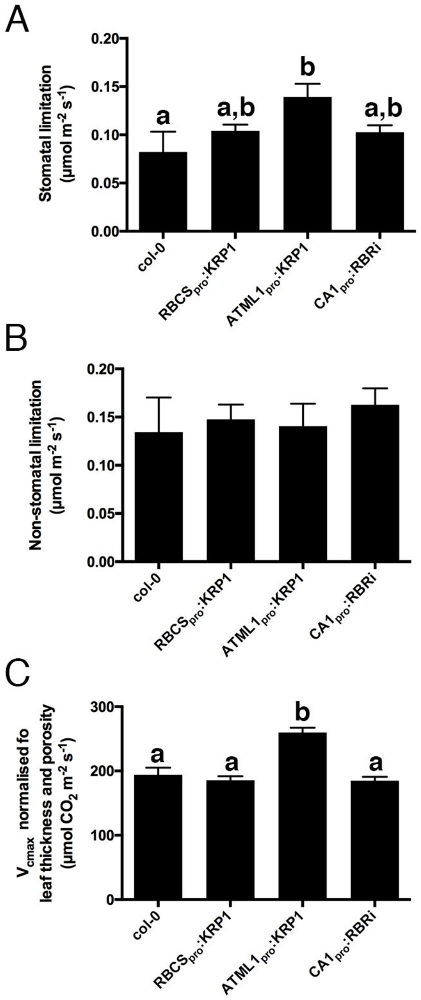

Sup Fig. 4

Analysis of stomatal and non-stomatal limitation

(A) Stomatal limitation **(B)** non-stomatal limitation and **(C)** V_{cmax} normalized for leaf thickness and porosity in col-0, $RBCS_{pro}$:KRP1, $ATML1_{pro}$:KRP1 and $CA1_{pro}$:RBRi leaves, as indicated. Values are means, error bars = sem. n > 6. Samples were compared with ANOVA followed by a post-hoc Tukey test. Columns indicated by identical letters within each analysis cannot be distinguished from each other at the 0.05 confidence limit.
