# Supplementary material for: Cell density and airspace patterning in the leaf can be manipulated to increase leaf photosynthetic capacity
Source: Plant J. 2017 Nov 15;92(6):981–94. doi: 10.1111/tpj.13727 (PMC5725688; doi:10.1111/tpj.13727)
Supplement: Supplementary file 5 — Figure S5. Generation and characterization of transgenic Arabidopsis. [file TPJ-92-981-s005.pdf]

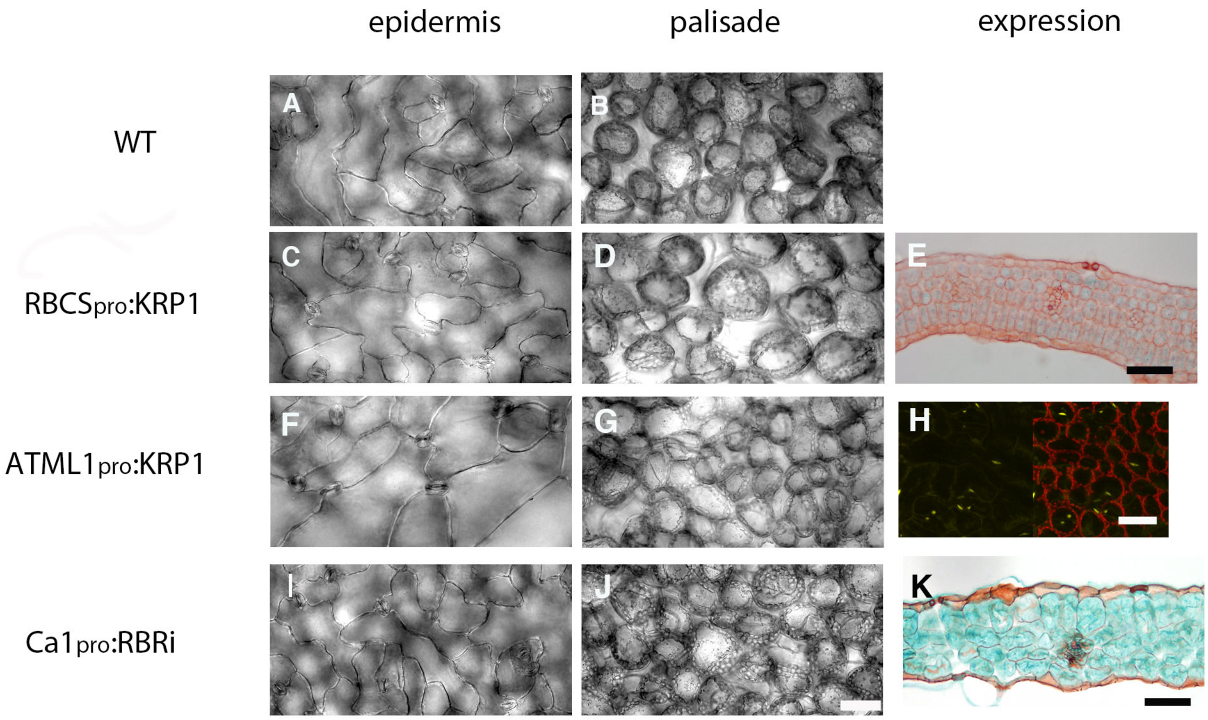

Sup Fig 5

Generation and characterisation of Arabidopsis genotypes

(A,C,F,I) DIC images of the upper epidermis of col-0, RBCSpro:KRP1, ATML:KRP1 and CA1pro:RBRi leaves.

(B,D,G,J) DIC images of the palisade of col-0, RBCSpro:KRP1, ATML:KRP1 and CA1pro:RBRi leaves.

(E) Cross-section through an RBCSpro:KRP1 leaf showing GUS signal (blue) in the mesophyll (counterstained with saffranin O).

(H) Confocal analysis of a ATML1pro:KRP1 leaf showing YFP signal (yellow) in the epidermal plane (left hand panel) and combined YFP (epidermis) and chlorophyll autofluorescence (red) in palisade cells (right hand panel).

(K) Cross-section through a CA1pro:RBRi leaf showing GUS signal (blue) in the mesophyll and limited signal in the epidermis (counterstained with saffranin O). Scale bars = 50 µm except for E, H, K = 100 µm.
