# Supplementary material for: Cell density and airspace patterning in the leaf can be manipulated to increase leaf photosynthetic capacity
Source: Plant J. 2017 Nov 15;92(6):981–94. doi: 10.1111/tpj.13727 (PMC5725688; doi:10.1111/tpj.13727)
Supplement: Supplementary file 7 [file TPJ-92-981-s007.docx]

**Supporting Information Legends**

**Sup Fig. S1**

**Epidermal cell size, stomatal density and stomatal conductance**

**(A)** Upper epidermal pavement cell area **(B)** area of leaf 8 at maturity **(C)** stomatal density in upper epidermis **(D)** mesophyll thickness and **(E)** stomatal conductance, *g*_s_, in *Col-0*, RBCS_pro_:KRP1, ATML1_pro_:KRP1 and CA1_pro_:RBRi leaves, as indicated. Values are means, error bars = sem. Values were measured in leaves from at least four (B) or at least five (A,C,D,E) independent plants. Samples were compared with ANOVA followed by a post-hoc Tukey test. Columns indicated by identical letters within each analysis cannot be distinguished from each other at the 0.05 confidence limit.

**Sup Fig S2**

**Analysis of light absorption and photosystem efficiency**

**(A)** Light absorptance **(B)** carboxylation efficiency, *CE* **(C)** the fraction of *PPFD* harvested by PSII, αβ **(D)** electron transport rate, J_400_ **(E)** the maximum efficiency of photosystem II, F_v_/F_m_ **(F)** the total pigments per area **(G)** initial/maximum quantum yield for CO_2_ fixation, *Y(CO_2_)*_LL_ and **(H)** total protein concentration per area in *Col-0*, RBCS_pro_:KRP1, ATML1_pro_:KRP1 and CA1_pro_:RBRi leaves, as indicated. Values are means, error bars = SEM. For A, B, D, F, G n ≥3; C ≥ 5; E ≥ 6. Samples were compared with ANOVA, followed by a post-hoc Tukey test. Columns indicated by identical letters within each analysis cannot be distinguished from each other at the 0.05 confidence limit.

**Sup Fig. S3**

**Analysis of chloroplast and cell structure**

**(A,B)** Confocal images of palisade cells in (A) *Col-0* and (B) *ATML1_pro_:KRP1* leaves revealing chlorophyll autofluorescence in plastids (red).

**(C,D)** TEM images of a chloroplast from a palisade cell in (C) *Col-0* and (D) *ATML1_pro_:KRP1*.

**(E,F)** TEM images of cell wall from a palisade cell in (E) *Col-0* and (F) *ATML1_pro_:KRP1*.

Scale bar: A,B = 50 µm; C,D = 2 µm; E,F = 0.5 µm

**Sup Fig. S4**

**Analysis of stomatal and non-stomatal limitation**

**(A)** Stomatal limitation **(B)** non-stomatal limitation and **(C)** *V*_cmax_ normalized for leaf thickness and porosity in col-0, RBCS_pro_:KRP1, ATML1_pro_:KRP1 and CA1_pro_:RBRi leaves, as indicated. Values are means, error bars = sem. Values were measured in leaves from at least six independent plants. Samples were compared with ANOVA followed by a post-hoc Tukey test. Columns indicated by identical letters within each analysis cannot be distinguished from each other at the 0.05 confidence limit.

**Sup Fig S5**

**Generation and characterisation of transgenic Arabidopsis**

**(A,C,F,I)** DIC images of the upper epidermis of *Col-0*, RBCS_pro_:KRP1, ATML1_pro_:KRP1 and CA1_pro_:RBRi leaves.

**(B,D,G,J)** DIC images of the palisade of *Col-0*, RBCS_pro_:KRP1, ATML1_pro_:KRP1 and CA1_pro_:RBRi leaves.

**(E)** Cross-section through an RBCS_pro_:KRP1 leaf showing GUS signal (blue) in the mesophyll (counterstained with saffranin O).

**(H)** Confocal analysis of a ATML1_pro_:KRP1 leaf showing YFP signal (yellow) in the epidermal plane (left hand panel) and combined YFP (epidermis) and chlorophyll autofluorescence (red) in palisade cells (right hand panel).

**(K)** Cross-section through a CA1_pro_:RBRi leaf showing GUS signal (blue) in the mesophyll and limited signal in the epidermis (counterstained with saffranin O).

Scale bars = 50 μm except for E, H, K = 100 μm.

**Sup Table S1.** Physiology and imaging data.
